# Diagnosis of cerebral malaria: Tools to reduce *Plasmodium falciparum* associated mortality

**DOI:** 10.3389/fcimb.2023.1090013

**Published:** 2023-02-09

**Authors:** Pranavi Muppidi, Emily Wright, Samuel C. Wassmer, Himanshu Gupta

**Affiliations:** ^1^ Department of Infection Biology, London School of Hygiene and Tropical Medicine, London, United Kingdom; ^2^ Department of Biotechnology, Institute of Applied Sciences & Humanities, GLA University, Mathura, UP, India

**Keywords:** cerebral malaria, plasmodium falciparum, diagnosis, biomarkers, therapeutic avenues

## Abstract

Cerebral malaria (CM) is a major cause of mortality in Plasmodium falciparum (Pf) infection and is associated with the sequestration of parasitised erythrocytes in the microvasculature of the host’s vital organs. Prompt diagnosis and treatment are key to a positive outcome in CM. However, current diagnostic tools remain inadequate to assess the degree of brain dysfunction associated with CM before the window for effective treatment closes. Several host and parasite factor-based biomarkers have been suggested as rapid diagnostic tools with potential for early CM diagnosis, however, no specific biomarker signature has been validated. Here, we provide an updated review on promising CM biomarker candidates and evaluate their applicability as point-of-care tools in malaria-endemic areas.

## Background

Malaria, a blood-borne parasitic disease, is a devastating illness that caused 247 million cases globally in 2021, increasing from the 227 million reported in 2019, and still predominantly affecting African paediatric populations (WHO, 2022). Caused by apicomplexan parasites of the *Plasmodium* genus, the greatest burden is found in tropical and subtropical parts of the world; approximately fifty percent of the world’s population is at risk of infection (Mace et al., 2018). Symptoms are often mild (e.g., fever, headaches, and vomiting) in endemic areas where populations develop a degree of immunity to the parasite, leading to uncomplicated malaria (UM) and asymptomatic malaria (AM, who harbour malarial parasites, but manifest minimal clinical symptoms) (Mace et al., 2018; Gupta and Wassmer, 2021). However, in non-exposed populations this immunity fails to build up and individuals are more likely to develop severe malaria (SM). SM is a broad-spectrum sepsis-like syndrome defined by organ dysfunctions caused by the excessive production of inflammatory mediators and sequestration of infected erythrocytes within the host’s microvasculature (White et al., 2013; WHO, 2014). This results in a range of complications, organ dysfunction, and systemic inflammation, which include cerebral malaria (CM), severe anaemia, acute respiratory distress syndrome (ARDS), intestinal injury (gut leak) and acute kidney injury (AKI) (Wassmer et al., 2015; Ouma et al., 2020; Namazzi et al., 2022; Ngai et al., 2022). CM is an often-fatal form of SM: it requires immediate intervention, has a mortality rate up to 30%, and long-term residual neurological complications. Some studies showed long-term sequelae/poor outcomes in 25-50% of survivors (WHO, 2014; Bruneel, 2019; Langfitt et al., 2019). Children under five are disproportionately affected, accounting for approximately 77% of deaths worldwide in 2020 (WHO, 2021). Children under five with CM are predominantly seen in Africa, as the malaria transmission intensity is high in sub-Saharan Africa, leading to the development of antimalarial immunity during childhood. In contrast, CM cases are mostly reported in older children and adults in Southeast Asia, where malaria transmission is seasonal, irregular, and do not allow the generation of a robust immunity (Sahu et al., 2015). Additionally, while CM is typically reported in combination with metabolic acidosis and/or severe anaemia in African children, it is frequently accompanied by lung, liver, and kidney dysfunction in Asian adults, resulting in ARDS, jaundice, and AKI, respectively (Wassmer et al., 2015). It is still unclear what causes these unique clinical traits in these two age and geographically distinct groups. While *Plasmodium falciparum* (*Pf*) is responsible for the highest number of malarial deaths, *Plasmodium vivax* (*Pv*) can also cause fatal disease. Previously thought to be relatively benign, *Pv* is now recognised to trigger potentially debilitating and life-threatening complications, although to a lesser degree than *Pf* (Gupta et al., 2015a; Gupta et al., 2016; Anvikar et al., 2020).

## Host response

**Figure 1:**
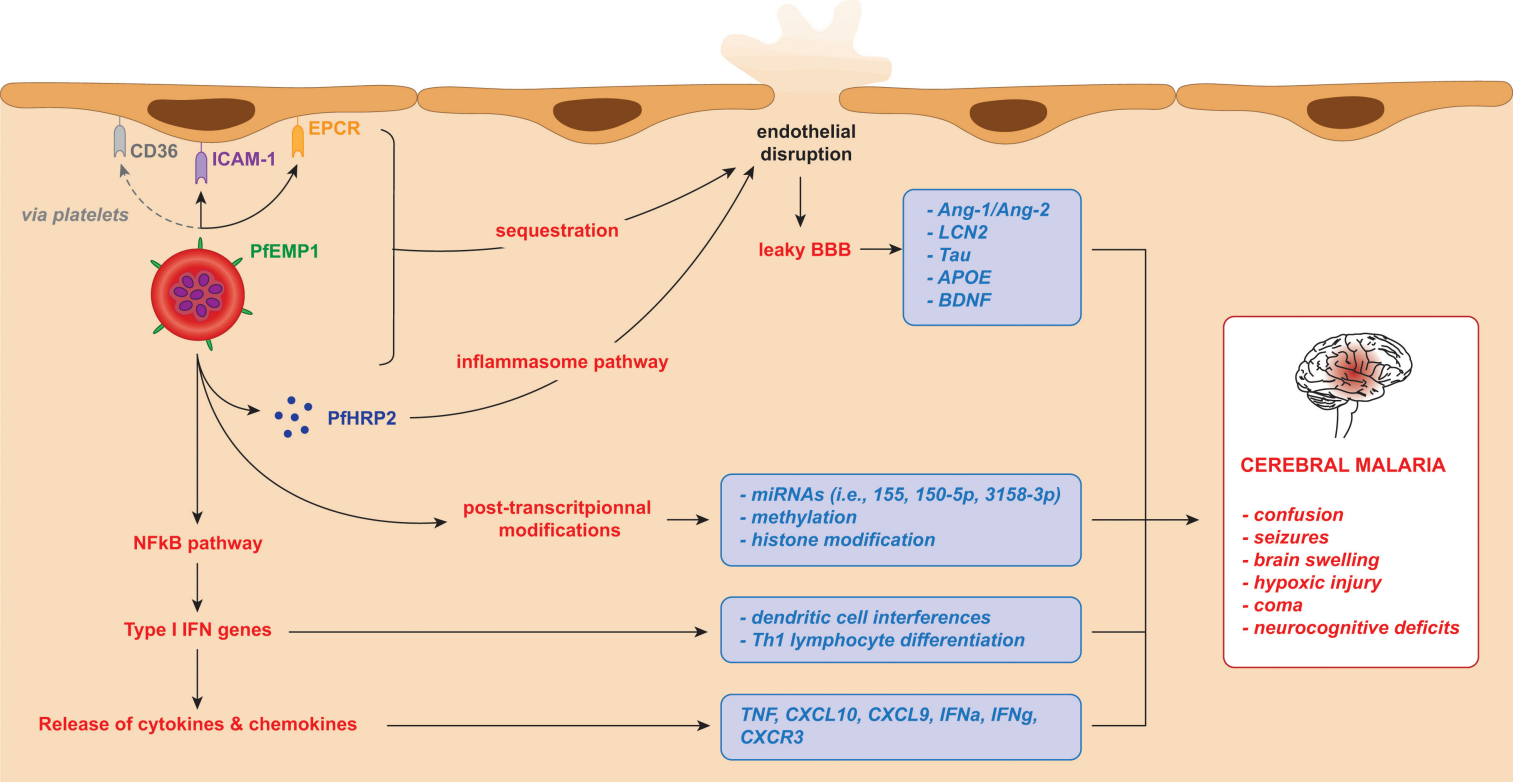
iRBC adhesion and subsequent release of potential biomarkers for cerebral malaria in *Plasmodium falciparum* infection. The cytoadherence of infected red blood cells triggers the release of parasitic and host components leading to a cascade of inflammatory mechanisms. In turn, these result in brain microvasculature endothelial disruption and clinical manifestations of cerebral malaria. These factors, listed in the blue boxes, can be considered as biomarkers of cerebral malaria.

During the blood-stage of *Pf* infection infected red blood cells (iRBCs) rupture, releasing parasitic components including3glycosylphosphatidylinositol anchors, haemozoin, and immunostimulatory nucleic acids (Gazzinelli et al., 2014). These components are detected by toll-like, retinoic acid-inducible gene I-like, and nucleotide-binding oligomerisation domain-like receptors on membranes and in the cytosol of host immune cells, triggering NFκB translocation to the nucleus, and activating type 1 IFN pathway genes in immune cells (Gazzinelli et al., 2014; He et al., 2020). This induces: IL-12 to promote the release of IFNγ by NK cells, CD4 and CD8 T cells; differentiation of TH1 lymphocytes; and interference with dendritic cell roles (Miller et al., 2014). Chemokines such as C-X-C motif chemokine ligand 10 (CXCL10) are also released to recruit NK cells (Wilson et al., 2011). The recognition of *Pf* infection by pathogen-associated molecular patterns (PAMPs) and damage-associated molecular patterns (DAMPs) inititate pro-inflammatory cascades, resulting in the release of cytokines (IL-1α, IL-1β, IL-6, IL-10, CXCL10, IFNγ and TNF) (Kwiatkowski et al., 1990; Mshana et al., 1991; Day et al., 1999; Lyke et al., 2004; Armah et al., 2007; Jain et al., 2008; Gazzinelli et al., 2014). The release of IFNγ then causes the release of further pro-inflammatory cytokines in a positive feedback loop, resulting in exacerbated inflammation and the upregulation of cell adhesion receptors on endothelial cells within the microvasculature of various organs, consequentially promoting iRBC cytoadherence (King and Lamb, 2015) (**Figure 1**). The IFN-I response is aimed at preventing replications within hepatocytes, but it appears insufficient against the vast number of merozoites released into the bloodstream upon iRBC rupture (Sebina and Haque, 2018; He et al., 2020). In addition to cytokine-mediated inflammation, systemic endothelial activation and dysregulation driven by endothelial and angiogenic factors contribute to organ failure and rapid disease progression (Higgins et al., 2016). The integrity of the blood-brain barrier (BBB) is also compromised by endothelial dysregulation mediated by parasite proteins such as *Pf* histidine-rich protein 2 (PfHRP2) (Pal et al., 2016), a soluble parasite-specific protein released by iRBCs during schizont rupture (Desakorn et al., 2005); by proinflammatory cytokines such as TNF and IFNy (Barker et al., 2017); through cytoskeletal remodelling caused by PfEMP1 and ICAM-1 interaction (Ramachandran and Sharma, 2022); and CD8^+^ T cell-mediated cytotoxicity (Riggle et al., 2020).

## iRBC adhesion

Healthy cerebral endothelium regulates platelet aggregation and coagulation, controls permeability, and prevents leukocyte adhesion *via* downregulation of adhesion molecules. The strong inflammatory response triggered by *Pf* infection interferes with these functions (Moxon et al., 2009).

iRBCs harbouring *Pf* parasites express *Plasmodium falciparum* erythrocyte membrane protein 1 (PfEMP1), which is synthesised within the erythrocyte during the blood stage of infection, exported on to its surface and plays an essential role in parasite adhesion and immunopathogenesis (Jensen et al., 2020). *Pf* uses the 60 *var* gene repertoires to switch between different PfEMP-1 variants, thereby facilitating immune evasion (Fairhurst et al., 2012). Selection pressures driving the parasite to improve receptor binding affinities while maintaining immune evasion have led to various specific encoded domains that interact with specific host endothelial receptors. This process is responsible for organ tropism, as different PfEMP1 subsets preferentially bind to host receptor molecules with specific domains (Montgomery et al., 2007). For example, PfEMP1 binds endothelial protein C receptor (EPCR) *via* structural elements named CIDRα1 domains (Lau et al., 2015; Kessler et al., 2017). Increased binding of PfEMP1 to EPCR *via* CIDRα1 is seen in SM (Jespersen et al., 2016; Bernabeu and Smith, 2017; Duffy et al., 2019) and may also be implicated in CM. Furthermore, a transcript analysis showed that CIDRα1 and parasite biomass are both strong indicators of disease severity (Bernabeu et al., 2016).

Host cell receptors such as intercellular adhesion molecule 1 (ICAM-1) and EPCR are expressed on the surface of endothelial cells, and when inflammation is triggered, TNF upregulates the expression of ICAM-1. PfEMP1 on the surface of iRBCs interacts with these receptors (Jensen et al., 2020), causing the iRBCs to adhere to the endothelial lining and sequester in the blood vessels of vital organs such as the brain, lungs, kidney, liver and placenta (**Figure 1**). This cytoadherence has been linked to SM and to the process of sequestration of iRBCs in the microvasculature of the host’s vital organs (Turner et al., 2013). Chemokines such as CXCL10 are secreted by cerebral microvasculature endothelial cells, reorganising tight junction proteins to increase BBB permeability, and allowing access to previously immune-privileged tissues (Tunon-Ortiz and Lamb, 2019).

Another form of cytoadhesion and suspected contributor to CM pathogenesis is ‘rosetting’, an aggregation of uninfected RBCs to iRBCs *via* the complement receptor-1 (CR1), potentially causing further vessel blockage and inflammation (Rowe et al., 1997). However, this phenomenon has only been demonstrated *in vitro*. Rosetting has been shown to be higher in SM compared to UM (Doumbo et al., 2009). Additionally, ‘clumping’, the platelet-induced formation of iRBC clumps within vessels, is also strongly associated with SM and its complications, such as CM (Pain et al., 2001; Wassmer et al., 2008). The combination of inflammation, sequestration, and dysregulated coagulation in CM act as a triple threat, contributing to overwhelming inflammation (Moxon et al., 2009).

## CM diagnosis, neuroimaging and limitations of diagnosis

The clinical definition of CM, as described by the World Health Organization (WHO), is a syndrome characterised by coma at least 1 hour after termination of a seizure or correction of hypoglycaemia, asexual forms of *Pf* parasites on peripheral blood smears, and no other reasoning for the coma (WHO, 2000). The latter is assessed by verbal and motor responses and is rated on the Blantyre coma score for pre-verbal patients such as infants (CM if < 3) or Glasgow coma score for verbal patients (CM if < 11) (WHO, 2014).

Current diagnostic measures for CM remain inaccurate. This was highlighted by Taylor et al., who demonstrated that 23% of a cohort of Malawian children clinically diagnosed with CM were found to have been misdiagnosed upon post-mortem examination (Taylor et al., 2004). This was supported by Makani et al. in 2003, who found that CM was over diagnosed (38%) when assessed by clinician’s judgement prior to consideration of available data, compared with diagnosis by fulfilment of the WHO criteria (1%) (Makani et al., 2003). A recent study from our team demonstrated that up to 20% of patients with non-CM, according to the WHO criteria, present magnetic resonance imaging (MRI) signatures associated with CM (Sahu et al., 2021b), confirming that it is a disease with a wide spectrum, with coma at one end of it (Mohanty et al., 2022).

Although full autopsies have been instrumental in advancing our understanding of CM pathogenesis, they have limited applicability in resource-limited settings, and novel live imaging techniques can provide critical information during life, especially by allowing the comparison of patterns associated with survival versus death. Relatives and guardians may be reluctant to authorise these procedures, particularly in areas with cultural or religious objections to post-mortem analyses. Furthermore, samples from deceased patients may not accurately reflect their state during life and could be influenced by agonal events. Therefore, there is a need for techniques allowing accurate early diagnosis in living patients.

Neuroimaging can increase confidence in the clinical diagnosis of CM by identifying pathological patterns (Sahu et al., 2021b). It offers a non-invasive, real-time approach to visualising brain changes, bypassing many of the challenges that exist with post-mortem diagnosis through autopsy and allowing diagnosis in living patients. Imaging-based identification of CM-associated patterns circumvents the pitfalls of the broad diagnostic criteria used clinically. Computerised tomography (CT) scanning has been used in several studies with varying results, likely due to limited sample sizes, differing timepoints through disease progression and inclusion of non-malarial patients (due to inaccurate diagnosis) (Mohanty et al., 2014).

Previous research studies have confirmed the occurrence of cerebral oedema in CM but did not conclude that it was the cause of death (Looareesuwan et al., 1983; Newton et al., 1994). However, poor prognosis of CM is closely associated with presence of cerebral oedema, found in 63% of patients (Mohanty et al., 2011). In one study from India, no patients with normal CT scans (i.e., showing no cerebral oedema, and with median Glasgow coma score of 10) died from the infection (Patankar et al., 2002). This cerebral swelling is likely multifactorial, caused by: (1) increased blood flow in the brain as a result of anaemia, (2) seizures; (3) fever; (4) sequestration of iRBCs; and (5) endothelial damage due to the overwhelming inflammation triggered by the host in response to *Pf* infection (Mishra and Newton, 2009). Sequestration contributes to endothelial cell activation, congestion within vessels, and reduced perfusion of tissue (Seydel et al., 2015), which, along with interactions between platelets and iRBCs, is suspected to cause the breakdown of the BBB. This injury allows excess fluid to accumulate within the brain parenchyma, leading to cerebral oedema (Idro et al., 2010).

The use of MRI in malaria research studies is relatively new and was limited to single case studies for a long time (Bellazreg et al., 2021; Schubert et al., 2021), before gradually becoming more available in endemic areas. A study of 24 adults demonstrated that brain swelling and increased volume is common in CM due to iRBC sequestration in the brain (Looareesuwan et al., 1995). Since its inception, MRI technology has improved greatly. A 2012 milestone trial of 120 Malawian children found that children with CM and retinopathies (Ret^+^CM), the latter consisting of whitening, vessel alternations, haemorrhages, papilledema, and cotton wool spots used to differentiate between malarial and non-malarial coma (Beare et al., 2006), presented numerous findings when compared with retinopathy-negative (Ret^-^) children who were comatose due to non-malarial reasons. These included: lesions of the basal ganglia; cerebral oedema; brainstem abnormalities; and changes to the corpus callosum and cerebellum (Potchen et al., 2012). Most of these features aligned with those previously seen in CT studies and intracranial pressure measurements in Kenyan children (Newton et al., 1991; Newton et al., 1994), and were distinct from the pathophysiology previously seen in adult patients (Millan et al., 1993; Cordoliani et al., 1998; Yadav et al., 2008). Several age-dependent brain changes identified by MRI on admission were linked to poor outcomes. In paediatric CM, severe brain swelling with brain stem herniation was associated with fatality (Seydel et al., 2015), a feature not observed in fatal adult cases (Mohanty et al., 2011; Maude et al., 2014). In the latter group, hypoxic injury evidenced by a decrease in apparent diffusion coefficient (ADC) values was associated with mortality (Sahu et al., 2021b). Furthermore, MRI studies in patients with UM and severe non-cerebral malaria (SNCM) showed both increased and decreased ADC values in the SNCM cohort compared with healthy controls and showed that low ADC values (suggesting cytotoxic oedema) demonstrated CM-like hypoxic patterns even without deep coma, and that high ADC values (suggesting mild vasogenic oedema) were present in both SNCM and UM patients (Mohanty et al., 2022).

Unfortunately, confirmation of diagnosis by neuroimaging requires expensive imaging facilities to be set up and accessible, which is often not financially or logistically feasible in malaria-endemic regions. In addition, early malarial symptoms are nonspecific and overlap with bacterial or viral infections, contributing to its late diagnostic and misdiagnosis. ‘Triage tools’ similar to those of septic children discussed elsewhere (Leligdowicz et al., 2021), may also allow earlier diagnosis and treatment of SM (including CM), once such prognostic biomarkers are identified. Finding an effective biomarker could make CM diagnosis more straightforward, reliable, faster, and less costly. CM may be more widespread than initially thought, so identification of patients with CM features unacknowledged in the WHO criteria may decrease societal consequences such as long-term neurocognitive sequelae (Mohanty et al., 2022). True CM could also be differentiated from false CM (Taylor et al., 2004) - this cannot be solely done on the basis of retinopathy, given that no clear associations have been established in adults (Mohanty et al., 2017). Biomarkers reflecting prognosis and severity of disease may dramatically reduce neurological sequelae and also guide decisions regarding aggressive treatment as well as resource allocation, for example in ICU settings. As therapeutic options improve in the future, these prognostic biomarker-based tools can be deployed in malaria-endemic areas against falciparum malaria for an accurate diagnosis and save lives by providing the treatment on time. Here, we cover the current knowledge of host and parasite factors in biological fluids that have previously been investigated for their potential as CM biomarkers across patient age groups.

## CM biomarkers

### Parasite factors

PfEMP1 is a central protein in *Pf* infection, key to parasite adhesion and sequestration. It evades immune recognition by frequently switching the variant of the molecule expressed (Jensen et al., 2020). Its essential role in disease pathogenesis makes it a molecule of interest in terms of associations with SM; this is supported by the fact that EPCR-binding PfEMP1 transcript levels are higher in CM than in milder or asymptomatic cases (Shabani et al., 2017). However, it is still not a promising biomarker due to numerous variants making it a difficult target.

Transcriptomic studies have helped to identify the conserved EPCR-binding sequences associated with CM. PfEMP1 molecules that bind to EPCR have been shown to belong to two distinct groups of *var* genes; A and B [as identified by RT-qPCR using loci-specific primers for certain PfEMP1 domain types (Tembo et al., 2014)]. Furthermore, following characterisation of the dominant transcripts in children with SM, it was found that CIDRα1 domains were the only common feature and that these were linked to CM (Jespersen et al., 2016; Kessler et al., 2017; Mkumbaye et al., 2017; Sahu et al., 2021a). Another study revealed a specific upregulation of genes involved in pathogenesis, adhesion to host cell, and erythrocyte aggregation in parasites from patients with CM compared to parasites from asymptomatic carriers (Almelli et al., 2014). Remarkably, UPs A1, A2, A3, B1, DC8 and DC13 *var* genes were also predominantly found in CM-associated isolates (Almelli et al., 2014). Recently, differential expression analysis showed that distinct transcriptome profiles between parasites from CM and UM patients, 284 genes were upregulated and 267 were downregulated in CM parasites compared with UM. Numerous upregulated genes (for example: *eba175* and *ama-1*) involved in entry into host pathway reflects an increased invasion capacity of CM isolates. Finally, genes involved in adhesion, excluding variant surface antigens, were dysregulated, supporting the idea of increased cytoadherence capacity of CM parasites (Guillochon et al., 2022). *var* A gene transcripts and CIDRα1 domains may therefore be central to the development of an effective CM biomarker (**Table 1**).

**Table 1:** Promising host and parasite factors-based CM biomarkers identified in humans.

Biomarker	Sample type	Method	Cut-off	Reference
CIDRα1 domains	Parasites from host blood	Next generation sequencing	96% sequence identity	Kessler et al., 2017
CXCL10	Plasma	ELISA	>831.2 pg/ml	Erdman et al., 2011
CXCL10 and CXCL4 combined	Plasma	ELISA	NA	Wilson et al., 2011
Angiopoietin-2/1 ratio	Serum	ELISA	>3.47 in adults, >0.14 in paediatric	Lovegrove et al., 2009
Tau	Plasma	Single-molecule array detection (Simoa)	>6.43 pg/ml	Datta et al., 2021
BDNF	Plasma	ELISA	NA	McDonald et al., 2017
miR-3158-3p	Plasma	RT-qPCR	>1.08 REL	Gupta et al., 2021b

Relative expression levels; NA, not available.

### Host factors

Cytokines have been shown to be crucial to the pathophysiological processes leading to CM (Dunst et al., 2017). When measured in CSF, some cytokines (IL-1ra, IL-8, CXCL10, PDGFbb, MIP-1β, Fas-L, sTNF-R1, and sTNF-R2) were significantly elevated in a CM mortality group (Armah et al., 2007). Of these, CXCL10 was the only serum cytokine to be independently associated with mortality (Armah et al., 2007).

While Type-I IFNs have been explored in diagnosis of other diseases, they are yet to be investigated as biomarkers in the context of CM, despite their clear relevance, notably in experimental models. Murine models with knockouts for the IFNα receptor showed partial protection from experimental CM post-infection by mechanism of reduced T-cell-associated cytokines including CXCL9 and CXCL10, thus highlighting the importance of IFNα in CM development (Palomo et al., 2013). Both CXCL9 and CXCL10 share the C-X-C chemokine receptor type 3 (CXCR3) which is present on various immune cells including NK, T, and NKT cells (Vazirinejad et al., 2014; Tokunaga et al., 2018). They recruit these cells to sites of immune compromise following recognition of *Pf* pattern recognition receptors. CXCL10 is produced by many cells, including endothelial cells, hepatocytes, and astrocytes (Park et al., 2006; Hassanshahi et al., 2007; Vazirinejad et al., 2014), and is implicated both in recruitment of cells to the original site of infection, but also across the BBB and into the brain, inducing CM progression.

Murine *P. berghei* ANKA studies have demonstrated that CXCL9, CXCL10 and CXCR3 are all required for CM development in experimental models, and that levels were increased in both serum and CSF (Campanella et al., 2008). Other murine models have shown that CXCR3 deficiency reduces CXCL9/10 mechanisms and protects mice from CM (Miu et al., 2008b). Despite this data, neither of these cytokines have been used as diagnostic markers in CM, although they have been explored in other diseases such as multiple sclerosis and trypanosomiasis (Balashov et al., 1999; Hainard et al., 2009). These cytokines could be promising rapid diagnostic testing candidates, particularly given the independent correlation of CXCL10 with CM development and mortality in both African and Indian cohorts in serum and CSF samples (Armah et al., 2007; Jain et al., 2008) (**Table 1**).

IFNγ also plays a key role in regulating inflammatory immune responses to control *Pf* infection in the liver and blood stages. However, its strong pro-inflammatory characteristics can lead to exacerbation of symptoms and overwhelming inflammation (King and Lamb, 2015). Its potential as a CM biomarker is limited due to its release upon infection by numerous other pathogens and in varying severities of malarial infection, meaning it is not specific enough to the CM disease state to be a viable option (Mitchell et al., 2005; D'Ombrain et al., 2008). In 2006, Prakash et al. investigated clusters of cytokines and their ability to determine disease severity; they found that IFNγ-related clusters were associated with mild or severe malaria, but not specific to CM (Prakash et al., 2006). Another finding of this study included a cluster of TNF, TGFβ, IL-10, and IL-1β which significantly correlated with CM.

TNF is released along with other pro-inflammatory cytokines such as IL-6, IL-1 and Fas. Many studies have reported that CSF levels of TNF, Fas-L, sTNF-R1 and R2, IL-6 and IL-1ra are significantly higher in CM cases, however, this was not consistently replicated in serum (Armah et al., 2007; John et al., 2008). Plasma levels of soluble TNF receptors and TNF were found to be higher in CM patients than those with UM, but other conflicting studies show that in serum TNF cannot distinguish between SM and UM (Molyneux et al., 1993; McGuire et al., 1998; Awandare et al., 2006).

Despite these promising findings regarding pro- and anti-inflammatory cytokines, a full signature is yet to be identified. Additionally, the specific point of disease progression at which cytokines are measured may also be critical to their usefulness as biomarkers. For example, TNF is released particularly early in the inflammation cascade, and so may not be a realistic candidate since relying on its detection would require rapid diagnostic testing to be carried out soon after infection – this is unlikely to happen if TNF levels rise then fall again before patients experience symptoms. This has resulted in conflicting conclusions regarding the potential role of TNF as a CM marker; in 1989, Grau et al. found that serum TNF positively correlated to fatality, hypoglycaemia, hyperparasitaemia, severity of disease, and declined in recovered patients (Grau et al., 1989). However, in 2009, Lovegrove et al. showed that, although serum TNF was significantly higher in Thai adults with CM, it was unable to differentiate CM from UM in African children (Lovegrove et al., 2009). Further work investigating cytokine profiles and their associations to CM progression in various biological fluids would make cytokines a more feasible option as biomarkers.

Angiopoietin-1 (Ang-1) and Angiopoietin-2 (Ang-2) act as ligands binding to the receptor Tie-2 (de Jong et al., 2016). Under normal conditions, Ang-1 binds to Tie-2 to promote an anti-inflammatory environment and apoptosis. Infection by *Pf* initiates inflammation, which causes Ang-2 to be released into the bloodstream from the Weibel-Palade bodies, tipping the balance between Ang-1 and Ang-2 in the favour of Ang-2 (de Jong et al., 2016). Ang-2 outcompetes Ang-1 in binding to Tie-2, leading to decreased anti-inflammatory and anti-apoptotic effects (Page and Liles, 2013; de Jong et al., 2016). Conroy et al. found that both CM and SM patients had significantly lower Ang-1 and higher Ang-2 levels than UM patients, and that CM patients had greater Ang-1 levels than SM patients (Conroy et al., 2009).

In 2016, De Jong et al. conducted a systematic review which found that all studies investigating Ang-1 and Ang-2 levels showed associations between increased disease severity, decreased levels of Ang-1, and increased levels of Ang-2 (de Jong et al., 2016). The studies included in the review also demonstrated the ability of Ang-2/1 ratio to distinguish between categories of malarial severity (de Jong et al., 2016). Lovegrove et al. showed that the Ang-2/1 ratio is a promising biomarker to distinguish UM from CM (Lovegrove et al., 2009). Although it is clinically easier to distinguish UM from CM using WHO guidelines, Ang-2/1 ratio could be used for diagnosis confirmation. It may also have prognostic value – many patients presenting with UM develop CM after admission despite initiation of treatment (Mousa et al., 2020; Borgstein et al., 2022), and Ang-2/1 ratio may allow the identification of such patients (**Table 1**).

Dysregulation of the Ang-2/1 balance is more associated with malarial severity than with CM specifically; although elevated Ang-2 concentrations have been linked to ‘pure’ CM, this increased expression was not significant when differentiating between cerebral and non-cerebral infections (Prapansilp et al., 2013). Furthermore, immunostaining of Ang-1, Ang-2 and Tie-2 did not correlate with iRBC sequestration in the brain. The work was conducted on post-mortem samples, and so cannot reliably be applied to *in vivo* infections (Prapansilp et al., 2013).

A study reported that the balance of Ang-1 and Ang-2 was not an effective measure of distinguishing between SM and CM. Only Ang-1 demonstrated a significant predictive value, but the area under the curve (AUC) value was limited to 73.5% (Conroy et al., 2009). Nevertheless, measurement of Ang-1 and Ang-2 can help improve confidence in the diagnosis of CM when used in conjunction with other biomarkers such as CXCL10, sFlt-1, PCT, sTREM-1 and sICAM-1; in 2011, these biomarkers were used in combination with Ang-2 to predict mortality in Ugandan children with SM, and together showed 95.7% sensitivity and 88% specificity in differentiating fatal and non-fatal cases (Erdman et al., 2011). Ang-1 has also proved useful in differentiating between Ret^+^CM and UM patients as well as between patients with Ret^+^CM and non-malaria febrile illness with decreased consciousness with AUC of 96% and 93%, respectively (Conroy et al., 2010).

### BBB dysfunction

A critical component in development of inflammation of CM is microvascular leakage – the process by which increased permeability allows plasma proteins and cells such as leukocytes to pass through and emigrate to other tissues (Renia et al., 2012; Nishanth and Schluter, 2019). Dysregulation of the BBB is a pivotal component of the pathology of *Pf*-related brain injury (Tunon-Ortiz and Lamb, 2019). The key regulators of this process are the endothelial cells lining blood vessels and acting as a barrier for circulating macromolecules and immune cells (Renia et al., 2012). Inflammatory conditions disrupt these barriers to accommodate migration of necessary immune cells and inflammatory protein such as cytokines (Chen et al., 2018). Notably, there is evidence for similarities in markers across a spectrum of brain injuries and neurodegenerative disorders (NDs) which provide a sound basis for exploring CM specific brain markers. Furthermore, BBB disruption also results in neurological sequalae, thus would be beneficial in establishing both prognostic and diagnostic markers (Tunon-Ortiz and Lamb, 2019). The disruption of the cerebral endothelium could also be pivotal for the detection of early CM (Conroy et al., 2010; Page and Liles, 2013).

Lipocalins, a protein family involved in cell homeostasis, transport, and immune functions, have great potential in indicating BBB disruption and have been investigated as biomarkers of vascular dementia. The pathology of vascular dementia involves impaired blood flow to the brain, and therefore may mimic the hypoxaemia and reduced blood flow also seen in CM due to iRBC sequestration (Llorens et al., 2020), which likely causes endothelial disruption and increased permeability of the BBB. Specifically, lipocalin-2 (LCN2) has been shown to play a role in innate immunity and to be expressed greatly in the CNS under inflammatory conditions (Xiao et al., 2017), suggesting that LCN2 hold potential as an early marker of CM.

LCN2 were able to discriminate vascular dementia from Alzheimer’s disease with good sensitivity (82%) and specificity (87%) (Llorens et al., 2020). Similarly, it was able to differentiate between vascular dementia and other neurodegenerative diseases with 78% of sensitivity and 82% of specificity (Llorens et al., 2020). This ability to distinguish between similar disease groups with high accuracy makes LCN2 an attractive biomarker for CM, which is commonly misdiagnosed as similarly presenting diseases including intracranial haemorrhage, Reye’s syndrome, poisoning and rabies (Beare et al., 2011). Furthermore, ELISA testing for LCN2 can diagnose brain injury (Suk, 2016). Given the similarities in pathology between brain injury and CM development, in addition to the potential role of LCN2 in BBB breakdown and neurodegenerative diseases, the use of LCN2 as a marker for CM is promising.

Whilst lipocalins have been investigated in the role of hemozoin production by *Plasmodium* and of neutrophil activation (Mohammed et al., 2003; Matz et al., 2020), a specific lipocalin in CM pathogenesis has not been widely researched. Its diagnostic ability in other neurodegenerative diseases marks it as an area of exploration, particularly through proteomic studies in fatal CM. Serum LCN2 has been found to be elevated in plasma in patients with fatal CM, particularly in adults, and this elevation was associated with decreased brain ADC on MRI (Sahu et al., 2021b). LCN2 may be particularly promising if it can be measured *via* antibody-based lateral flow assays for use in simple, cost-effective methods, with potential for scaling into point-of-care tools.

The tau protein is involved in microtubule assembly and stabilisation. During brain injury or degeneration, its expression is increased, and this has recently been shown in CM (Weingarten et al., 1975; Buee et al., 2000; Morris et al., 2011). Animal models have demonstrated that this increased expression could make tau protein a useful biomarker of brain injury, specifically axonal damage (Bi et al., 2017). CSF tau levels were significantly elevated in children with CM compared with either malaria with prostration or malaria with seizures but normal consciousness, which suggests axons are more vulnerable to damage in childhood and could potentially explain the greater incidence of sequelae in children (Medana et al., 2007). Although immunoassays have shown a significant increase in tau levels in CSF of Vietnamese adults diagnosed with SM, this did not hold true for CM patients, but there was a significant association between CSF tau level and length of coma (Medana et al., 2005). Associations were also found between CSF tau and levels of other markers linked to severe disease, such as plasma creatinine, blood urea nitrogen, and serum glutamic oxaloacetic transaminase. Importantly, statistical significance was still achieved when age-group outliers were removed from analyses (Medana et al., 2005).

These findings were confirmed by Datta et al. in 2020: they found that higher CSF tau levels correlated with younger age, increased disease severity (evidenced by low glucose, kidney injury, and prolonged coma death) and an elevated CSF-to-plasma albumin ratio, a marker of BBB breakdown (Datta et al., 2020). In 2021, they demonstrated that plasma tau is also raised in children with CM and is linked to mortality and long-term neurocognitive impairment in children under five (Datta et al., 2021).

Despite these promising results, CSF-based biomarkers present drawbacks. There are often discrepancies between CSF and plasma marker levels. CSF sampling can also be time-consuming, and sequential samples cannot be taken from children as this exposes them repeatedly to the associated risks, such as bleeding and subdural haematomas, infection, and damage to surrounding structures. Additionally, CSF sampling requires training and equipment that may be difficult to access locally in community clinics. Furthermore, CSF can act as an optimum medium for growth of other bacterial or fungal infections that could bias the results. Refrigerating samples is not sufficient; they must be frozen at sub-zero temperatures, creating another barrier to the accessibility of the testing method in endemic areas (Teunissen et al., 2009). Moreover, cultural acceptance of lumbar puncture remains limited in some endemic areas, and represents a major hurdle to obtain CSF (Thakur et al., 2015; Elafros et al., 2022). However, serum/plasma tau levels may be a viable alternative to CSF levels. In 2020, Jain et al. demonstrated that detection of serum tau levels increased with disease severity, and that detection was much higher in CM patients, particularly non-survivors, than non-CM patients (Jain et al., 2020; Datta et al., 2021). This work suggests that serum tau levels detected by ELISA could be a viable alternative diagnostic marker in endemic regions (**Table 1**). Remarkably, technological advances have allowed the detection of plasma/serum markers at femtogram levels [i.e., Simoa (Sanchez-Valle et al., 2018; Zeitlberger et al., 2018)] and that will revolutionise biomarker identification in the blood. However, there’s the caveat of the transfer to lateral flow assays.

In recent years, anti-TNF treatments have been utilised for the treatment of neurodegenerative diseases. Research into these has led to the discovery of potential links between TNF and brain proteins such as apolipoprotein E (ApoE) (Clark and Vissel, 2018). TNF can cross the BBB, leading to pro-inflammatory conditions associated with brain injury and neurodegeneration; however, it is known to be a poor correlate for CM severity (Turner et al., 1998; Gimenez et al., 2003; Lovegrove et al., 2009). Anti-TNF treatments have also proven ineffective (Kwiatkowski et al., 1993; van Hensbroek et al., 1996). However, serum apolipoprotein E (ApoE) shows potential as a CM biomarker. In the mouse model, deletion of *ApoE* protected animals against CM, reduced parasite sequestration within the brain, and prevented BBB disruption and vascular leakage (Kassa et al., 2016). Despite limited evidence of diagnostic value, ApoE is easily measured in serum, and upon further study, may prove useful to detect CM-associated BBB impairment in conjunction with other brain factors.

Another potential marker implicated in neurodegenerative diseases is the brain-derived neurotrophic factor (BDNF). In a cohort of Ugandan children, lower levels of BDNF on admission were associated with more severe disease, including CM, and higher chance of disability or death (McDonald et al., 2017). This highlights its potential as a prognostic marker of disease (**Table 1**), but further studies are needed for its validation.

### Genetic signatures

Evidence indicates that host genetics can regulate *Pf* infection, specifically in severe, life-threatening manifestations of the disease (Jallow et al., 2009; Gupta et al., 2013; Saadi et al., 2013; Fernandes et al., 2015; Gupta et al., 2015b; Mukhi et al., 2020). During human evolution, there are classical examples of genetic variations that have occurred to provide resistance against malaria, including *i)* sickle-cell trait; *ii)* thalassemia; *iii)* glucose-6-phospahte dehydrogenase (G6PD) deficiency; and *iv)* Duffy antigen deficiency (Hedrick, 2011). Therefore, single nucleotide polymorphisms (SNPs) could explain CM predisposition, especially in malaria-related genes that are involved in immunological responses and cell receptors. *TNF* promoter region SNPs have been associated with CM susceptibility in several African and Asian populations (Gimenez et al., 2003; Hananantachai et al., 2007). SNPs present in *CR1* have been shown to contribute to protection against CM both in Indian and Thai populations (Teeranaipong et al., 2008; Panda et al., 2012). Similarly, SNPs present in *ABCA1* (Sahu et al., 2013), *TIM1* (Nuchnoi et al., 2008), *ICAM-1* (Fernandez-Reyes et al., 1997), *HO-1* (Takeda et al., 2005), *PECAM-1/CD31* (Kikuchi et al., 2001) and *CD36* (Omi et al., 2003) genes have been associated with CM susceptibility. Jallow et al. in 2009 demonstrated the association between CM susceptibility and SNPs present in several chromosomal regions and genes (Jallow et al., 2009). These genetic signatures are promising and can be used as prognostic biomarkers to identify and treat patients at the risk of developing CM. However, the genetic background variations in each ethnic population adds to the differences in levels of disease predisposition and severity. Thus, further studies with large sample sizes are required to confirm these results in different geographic populations, as well as to determine whether these genetic signatures will be suitable for point-of-care testing. The recent advancement in DNA sequencing technology now allow SNP analysis in a large population of individuals through targeted sequencing of specific gene sets and circumvents the financial challenges of whole human genome sequencing (Bansal et al., 2010; Tabata et al., 2022).

### Thrombocytopenia

Platelets have long been implicated in the pathogenesis of CM. They accumulate in the cerebral microvasculature in fatal paediatric CM cases (Grau et al., 2003), bind to endothelial cells, providing previously absent PfEMP1 receptors (Wassmer et al., 2004), and form clumps with iRBCs (Pain et al., 2001; Wassmer et al., 2008). Through these three mechanisms, they have been suspected to aggravate microvascular obstruction during CM (Wassmer et al., 2011). Conversely, studies have also shown that platelets can block the growth of parasites *in vitro* (McMorran et al., 2009; McMorran et al., 2012), suggesting that they are activated as part of the innate immune response to the infection. However, when parasitemia increases, they become active players in CM pathogenesis (Wassmer et al., 2011). Indeed, platelet-erythrocyte complexes leading to parasite killing were found to make up a major proportion of the total platelet pool in patients with malaria and may therefore contribute considerably to malarial thrombocytopenia (Kho et al., 2018). In turn, this platelet-mediated clumping of iRBCs may worsen the plugging of brain microvessels in CM, and severe thrombocytopenia in this context may become a protective strategy for the host (Wassmer et al., 2008). Thrombocytopenia has been associated with CM (Grau et al., 2003); platelets have been found in mouse studies to play a negative role in CM, which may be reliant on the presence of *Plasmodium* infection *via* CD36-dependent interactions (Lou et al., 1997; Grau et al., 2003; van der Heyde et al., 2005; Wassmer et al., 2006; Srivastava et al., 2008; McMorran et al., 2009). Thrombocytopenia is common in all types of malaria (Naing and Whittaker, 2018) and has also been used as a diagnostic marker for the disease (Gebreweld et al., 2021). In children with falciparum malaria, thrombocytopenia has been shown to be a good predictor of disease severity and outcome (Gerardin et al., 2002). However, contradictory findings have been provided, implying that thrombocytopenia can only predict parasitaemia, not malaria severity (Ladhani et al., 2002; Arman et al., 2007). Furthermore, platelet sequestration within brain microvessels, increased production of pro-inflammatory cytokines, and disruption of the BBB are all hallmarks of CM brain lesions (Combes et al., 2006; Wassmer et al., 2006). Remarkably, CM children with retinopathy exhibited considerably lower platelet counts than those without (Chimalizeni et al., 2010), probably owing to immune destruction of circulating platelets, splenic pooling, reduced platelet lifespan, and accumulation of platelets in brain microvessels (Chimalizeni et al., 2010; Wassmer et al., 2011). Recently, the subgrouping of patients with CM revealed that severe thrombocytopenia was associated with increased parasite biomass, while moderate thrombocytopenia was associated with more Group A–EPCR *var* transcripts (Sahu et al., 2021a), suggesting that these elements could be investigated in combination with thrombocytopenia to determine CM prognosis, although further research is needed.

During platelet aggregation, CXCL4 is released from the alpha-granules of activated platelets. CXCL4 is involved in the regulation of haematopoiesis and angiogenesis, as well as the control of immunity and inflammation (Maurer et al., 2006). It has been shown that iRBC activates platelets and stimulates CXCL4 secretion (Srivastava et al., 2008). Platelet build-up in cerebral microvessels of CM patients suggests that platelets and CXCL4 may play a role in its pathogenesis (Grau et al., 2003). CXCL4 levels have been linked to acute human malaria (Essien and Ebhota, 1983) and in experimental CM (ECM) (Srivastava et al., 2008). TNF, a major proinflammatory cytokine linked to the development of CM, is stimulated by CXCL4 (Gimenez et al., 2003; Wilson et al., 2011). TNF also promotes platelet binding to brain microvasculature during ECM, implying that platelets are important in CM pathogenesis (von Zur Muhlen et al., 2008; Srivastava et al., 2010). According to a study, CXCL4 negatively drives immunological stimulation and monocyte activation in ECM (Srivastava et al., 2010). Another study found that significant increase in CXCL4 plasma levels is linked to CM (Wilson et al., 2011). This adds to the data suggesting CXCL4 is involved in CM immunopathogenesis (Srivastava et al., 2008; Srivastava et al., 2010) and may also be used as a diagnostic biomarker of CM alone and in combination of other molecules (**Table 1**). Remarkably, combination of CXCL4 and CXCL10 have been shown to predict risk of fatal CM (Wilson et al., 2011). Receiver operating characteristic (ROC) curve analysis demonstrated their ability to differentiate CM non-survivors from those with mild malaria (MM) (P<0.0001) and from CM survivors (P<0.0001), with an AUC of 100% (Wilson et al., 2011). However, further research with larger sample size in different populations is needed as this study only included 80 samples (16 healthy control, 26 mild malaria, 26 CM survivors and 12 non-survivors) from Madhya Pradesh, India.

### Transcriptomic signatures

The examination of genome-wide RNA expression, known as transcriptomics, is a method to study host and pathogen mechanisms involved in infectious diseases. Advances in technology and bioinformatics have allowed many transcriptomic in-depth analyses of *Plasmodium* species conducted *in vitro* and *in vivo* to understand the relationship between RNA expression and fundamental malaria biology, immunity, and pathogenesis, as well as to identify diagnostic and prognostic biomarkers (Lee et al., 2018a). A study reported 842 genes whose expression differed between patients with CM and MM. Five out of six CM patients and six out of six MM patients were accurately identified using differentially expressed genes using the support vector machine method. It was also shown that genes involved in immunological signalling pathways appear to play a role in the development of CM, according to functional enrichment analysis. These included BCR-, TCR-, TLR-, cytokine-, FcϵRI-, and FCGR- signalling pathways, and natural killer cell cytotoxicity pathways, which are involved in the activation of immune cells (Thiam et al., 2019). Additionally, whole-blood transcriptomes of Malawian CM children with Ret^+^CM and Ret^-^CM were compared. Upregulation of 103 gene sets, including cytokine, blood coagulation, and extracellular matrix (EM) pathways, was linked to Ret^+^CM. Neutrophil transcripts were the most elevated in Ret^+^CM patients. Activated neutrophils can influence a variety of host processes, including the EM, inflammation, and platelet biology, which could aid parasite sequestration (Feintuch et al., 2016). Pathways associated to coagulation, platelet activation, and cytokine signalling were also overrepresented (Feintuch et al., 2016), which aligns with other studies on coagulopathy and inflammation involving CM patients (Moxon et al., 2009; Moxon et al., 2015). In Ret^+^CM patients, plasma levels of TNF, neutrophil primary granule proteins, monocyte chemotactic protein 1, and IL-10 were higher. In contrast, higher concentrations of plasma type I IFN was associated with Ret^-^CM patients (Feintuch et al., 2016). Similarly, upregulation of type I IFN was associated with UM but not with CM in Malawian Children (Krupka et al., 2012). Mutations in the type I IFN receptor gene have been linked to protection against SM, and type I IFNs may control endothelial alterations to protect against iRBC sequestration have been demonstrated (Aucan et al., 2003; Khor et al., 2007). In the ECM model, type I IFN-treated mice showed improved survival, lower ICAM-1 expression in brain endothelial cells, and decreased serum TNF levels (Vigario et al., 2007; Morrell et al., 2011). However, a recent study demonstrated that type-I IFN levels correlated negatively with parasite load, suggesting that downregulation of type-I IFN with high parasite load ultimately increased severity (Lee et al., 2018b). Similarly, ECM-based studies demonstrated that type I and type II IFN signalling are enriched and upregulated in ECM compared to comparators (Sexton et al., 2004; Delahaye et al., 2006; Lovegrove et al., 2006; Lovegrove et al., 2007; Miu et al., 2008a; Berghout et al., 2013). These results suggest that although findings on type I IFN in CM patients remain contradictory, it still has an important role in CM.

Boldt et al. (Boldt et al., 2019) found strong repression of IFN beta-regulated genes and of genes with key roles in IFN signalling, of which IFNβ has emerged as a strong candidate for the treatment of CM (Franklin et al., 2011; Morrell et al., 2011). In addition, it appears that downregulation of several genes in CM patients may be a response to hypoxia, orchestrated by AhRF, GABP and HIF1 transcription factors. This correlated with hypoxia effects due to sequestration of iRBCs and vessel occlusion in CM children. Thus, improving perfusion to diminish hypoxic injury may be beneficial in children with CM (Beare et al., 2009; Boldt et al., 2019). Cabantous et al. (Cabantous et al., 2020) identified 538 differentially expressed genes between CM and UM patients. Pathway analyses revealed novel genes and biological pathways related to immune/inflammatory responses, erythrocyte alteration, and neurodegenerative disorders. Microarray analysis showed that CXCL10, IL12RB2, IL18BP, IL2RA, AXIN2, and NET expression levels were significantly lower in CM whereas ARG1 and SLC6A9 were higher in CM compared to UM. Upon validation using RT-qPCRs, all the selected genes showed significant changes between CM and UM consistent with those observed by microarrays (Cabantous et al., 2020). In addition, plasma protein levels of CXCL10 were significantly lower in CM than in UM while levels of IL-18 were higher. Remarkably, among children with CM, those who died from malaria complications generally had higher concentrations of CXCL10 and IFN-γ than those who recovered (Cabantous et al., 2020). Li et al. identified MBP, SAMSN1, PSMF1, SLC39A8, EIF3B, SMPDL3A, FABP5, SPSB3, and SHARPIN genes, which were associated with CM, and suggested that these genes may be good potential targets or immune modulators for novel therapeutic interventions of CM (Li et al., 2021). In addition, newly developed technologies like Olink can further help to identify protein signatures utilizing minimal sample volume with unrivalled specificity and sensitivity, as recently shown for active tuberculosis (Mousavian et al., 2022).

### Neutrophil extracellular traps

Formation of neutrophil extracellular traps (NETs) is one of the important innate strategies for killing pathogens. This is triggered when activated neutrophils degranulate, allowing neutrophil antimicrobial factors to enter the extracellular environment. NETs are web-like structures made up of highly modified chromatin and different antimicrobial granular proteins that can kill and neutralise several microbes (Brinkmann et al., 2004). NET formation can be influenced by several factors including crystal uric acid (a potent inducer of NETosis) (Hoppenbrouwers et al., 2017) and its precursor hypoxanthine, which is released upon iRBC rupture (Gallego-Delgado et al., 2014), and extracellular haem, a malarial DAMP (Knackstedt et al., 2019). Additionally, TNF and IL-8, which are increased during *Plasmodium* infections (Dunst et al., 2017), and immune cells stimulated by *Plasmodium* antigen produces H_2_O_2_ (Percario et al., 2012) have been shown to induce NETosis (Hoppenbrouwers et al., 2017). Similarly, CXCR4 and macrophage migration inhibitory factor (MIF) are required for NET release triggered by iRBCs (Rodrigues et al., 2020). Neutrophils impose strong immune pressure against PfEMP1 variants implicated in CM, selectively eliminating iRBCs expressing subsets of PfEMP1 with ICAM-1-binding properties (Zelter et al., 2022). Activated neutrophils were shown to be associated with CM, an autopsy study showed that neutrophils were rarely present in brain microvasculature of Malawian children (Feintuch et al., 2016). However, another study demonstrated that NETosis was strongly associated with iRBC sequestration in retinal capillaries of children who died from CM (Knackstedt et al., 2019). In the murine model of CM, neutrophils were also found to play a critical role in the pathogenesis of ECM (Chen et al., 2000). The release of matrix metallopeptidase (MMP) -8 and 9 is another sign for neutrophils activation. MMP-8 levels have been shown to be higher in plasmas obtained from malaria patients (Dietmann et al., 2008). In addition, MMP8 release within the retina has been associated with parasite sequestration in brain blood vessels of Malawian children clinically diagnosed with CM, with a median of 88% of capillaries containing MMP8, compared to 14% in those diagnosed clinically but without parasite sequestration (Georgiadou et al., 2021). This expression of MMP8 was also strongly linked with extravascular fibrinogen leakage, suggesting that MMP8 release may cause the vascular endothelial barrier disruption in CM, potentially precipitating fatal brain swelling. MMP-9 was found in brains from CM patients but not in brains of mice with non-CM. However, MMP-9 knockout had no significant effect on CM development (Van den Steen et al., 2006). Further studies examining NETs in clinical samples from patients with CM and in organs outside the brain in SM are warranted.

### microRNAs and methylation

The concept of using microRNAs (miRNAs) as biomarkers for severe disease in malaria remains relatively novel. miRNAs are small, highly evolutionarily conserved noncoding RNAs ranging from 18 to 24nt, which regulate gene expression, transcription and translation by interacting with mRNA post translation. They are detectable in plasma, serum, urine, and tissue, and have been shown to be highly stable in a wide range of biological fluids and samples of fixed tissues, which makes them exceptionally promising non-invasive biomarkers suitable for use in RDTs in regions with fewer healthcare facilities (Gupta and Wassmer, 2021; Sikka et al., 2022). miRNAs have been evaluated as biomarkers in several diseases so far, including infectious diseases, cancers, cardiovascular diseases and diabetes (Xu et al., 2014; Condrat et al., 2020; Nazarizadeh et al., 2020; Tribolet et al., 2020; Gupta and Wassmer, 2021; Gupta et al., 2021a). Meta-analyses of miRNA biomarker studies reported higher accuracy in diagnoses using miRNA clusters compared to single miRNA (Gao et al., 2020). This is promising, considering the clusters of miRNAs that have been identified in CM.

miR-155 has a key role in the pathogenesis of CM through dysregulation and compromise of the BBB and through T-cell functioning (Barker et al., 2017). Furthermore, murine studies have highlighted a number of biomarker candidates for CM, such as miR-19a-3p, miR-540-5p, miR-223-3p, miR-142-3p, miR-19b-3p, let-7i, miR-27a, miR-150, miR-146a, miR-193b, miR-205, miR-215 and miR-467a (El-Assaad et al., 2011; Cohen et al., 2018; Martin-Alonso et al., 2018). These miRNAs are associated with various CM-related pathways – inflammation, TGF-β, TNF signalling, monocyte sequestration in cerebral microvessels, and endocytosis (El-Assaad et al., 2011; Cohen et al., 2018; Martin-Alonso et al., 2018). Of the noted candidates, miR-146a-5p, miR-150-5p, miR-222-3p and miR-3158-3p were also linked to CM in a cohort of Indian patients (Gupta et al., 2021b). On further study, increased levels of miR-150-5p and miR-3158-3p were shown to correlate with fatal disease. By 30 days following treatment, miR-3158-3p levels were notably lower in the CM cohort who survived, greatly implying the specificity of the miRNA to CM. Finally, a positive correlation with hypoxia in adults’ brains, and a negative correlation with increase in volume in children’s brains, were both found with miR-3158-3p upon MRI (Gupta et al., 2021b). This suggests that miR-3158-3p production is lower in CM patients with increased brain volume upon admission (a feature linked to poor outcome in children) (Seydel et al., 2015; Sahu et al., 2021b). Conversely, patients with marked hypoxia on admission, a hallmark of fatal CM in adults, had increased levels of miR-3158-3p (Sahu et al., 2021b). Although the associations between this miRNA and MRI signs suggesting poor CM outcomes need further corroboration, these findings support the potential utility of miR-3158-3p in determining CM prognosis in both children and adults without the need for neuroimaging (Gupta et al., 2021b) (**Table 1**).

They have the potential to fulfil the requirements of good biomarkers in a number of ways: they are accessible through minimally invasive techniques such as blood tests and urine sample collection; they have been found to vary with disease severity, meaning they could be used to guide disease management; nucleic acid detection technology already exists and could be honed to develop malaria-detecting lateral flow assays, an approach already being explored in cancer (Gupta and Wassmer, 2021); and several miRNAs could be detected within one test to provide a more comprehensive picture of the patients’ condition.

In addition to non-coding RNAs, there are two other main mechanisms of post-translational modification: methylation, the critical regulation of gene expression *via* targeting of cytosine-guanine motifs where numerous promoters lie; and histone modification, the alteration of the tail ends of histone proteins (Chhabra, 2015; Cross et al., 2019).

Methylation and demethylation of promoter regions allow genes to be turned on or off (Gupta H. et al., 2017). This can inhibit miRNA transcription by methylation of specific CpG sites. Equally, miRNAs can prevent methylation by blocking the activity of DNA methyltransferases, which are involved in the addition of methyl groups. Some infectious agents have evolved to manipulate this process to promote survival. For example, *Mycobacterium tuberculosis* alters the function of the IL-12B gene (Chandran et al., 2015). DNA methylation-based panels have been used for the prognosis and diagnosis of patients with breast cancer (Liu et al., 2020), in some cases outperforming other biomarkers in prediction of survival. Although methylation of inflammatory genes has been widely explored, there is limited knowledge of the role of this process within the context of malaria (Gupta H. et al., 2017; Arama et al., 2018).

The similarities in disease progression between sepsis and CM opens new avenues to identify CM-specific aberrantly methylated genes as in sepsis. Several specific regions so far have been associated with severe sepsis, for example, DNA methylation of a specific binding site for Nf-κβ was found to be independently associated with increased risk of death in sepsis patients (Rump et al., 2019). Likewise, another study identified a correlation between specific methylation changes in monocytes of sepsis patients, IL-10 and IL-6 levels, and organ dysfunction (Lorente-Sorolla et al., 2019). Hypermethylation occurred in genes for immune and inflammatory processes, including MAPK and NF-κB signalling, and chemokine–cytokine pathways; hypomethylation occurred in genes involved in IFNγ signalling and phagocytic vesicles. It was also shown that organ dysfunction was linked to changes in DNA methylation.

Elevated IL-1β and IL-6 in Alzheimer’s disease echo the neuroinflammation seen in sepsis. One 2017 study found hypomethylation in the IL-1β promoter region and higher levels of methylation in IL-6 were linked to disease (Nicolia et al., 2017). Another more recent study by Altuna et al. found approximately 118 differentially methylated positions (DMPs) in the hippocampus of Alzheimer’s patients, which were significantly correlated with neurogenesis (the process by which neural stem cells give rise to new neurons), including tau burden (Altuna et al., 2019).

The involvement of DMPs in neurogenesis and development suggest a role for them in early Alzheimer’s disease development, highlighting them as effective diagnostic biomarkers. Given the involvement of tau in CM pathogenesis, DMPs may be a potential area for consideration in CM biomarkers. Furthermore, it has been shown that both ApoE and BNDF promoters were subject to hypermethylation in Alzheimer’s patients (Wang et al., 2008; Chang et al., 2014).

Aberrant methylation patterns are seen in a vast number of genes associated with CM. Positively, there is agreement between studies, particularly those investigating genes associated with inflammatory pathways. If specific genes involved in the pathogenesis of CM were to be identified, gene-specific methylation analysis may be an attractive method for diagnosis. Given the conclusions of studies into sepsis and Alzheimer’s disease, it would be beneficial to explore the methylation status of tau, ApoE, BDNF, IL-1 and IL-6 alongside more CM-specific genes such as CXCL10.

Practically, when beginning to identify a wide range of methylation clusters (multi-gene analysis), an epigenome-wide approach is preferred. However, once specific genes are identified, single-gene analysis (which is relatively rapid and cost-effective) can be performed to aid diagnosis of CM (Sant et al., 2012). Overall, this area remains greatly under-researched in CM and further studies identifying panels of methylation targets could present advances in the search for a suitable diagnostic tool.

### Metabolites

Metabolomics is another avenue of exploration to identify metabolites with the potential to act as markers in diagnosis of *Plasmodium* infections. Several attempts have been made in rodent models (Ghosh et al., 2011; Ghosh et al., 2012; Ghosh et al., 2013; Ghosh et al., 2016; Srivastava et al., 2016) and humans (Lopansri et al., 2003; Medana et al., 2003; Pappa et al., 2015; Alkaitis et al., 2016; Sengupta et al., 2016; Holmberg et al., 2017; Gupta S. et al., 2017) to demonstrate the potential of metabolomics as a tool to differentiate malaria severity, ranging from asymptomatic to CM. A study reported a low plasma arginine concentration in children with CM and decreased nitric oxide (NO) production (Lopansri et al., 2003). Similarly, CM patients exhibited low plasma glycoproteins (Sengupta et al., 2016). In contrast, CSF NMDA (*N*-methyl-D-aspartate)-receptor antagonist kynurenic acid and kynurenine were elevated in children with CM, indicating an inhibition of glutamatergic and cholinergic signalling that may lead acute to prolonged coma (Holmberg et al., 2017). Tryptophan catabolites are of interest given that elevation of CSF levels of kynurenine and picolinic acid have been reported in CM patients. In addition, picolinic acid was also shown to be significantly associated with hyperparasitaemia (Medana et al., 2003). Another study reported a series of significant changes in levels of kynurenate, indolepropionate, glutamate, arginine and glutamine molecules that could impact neurologic function during CM (Gupta S. et al., 2017). Children with CM were also found with depleted plasma arginine, ornithine, and citrulline levels (Alkaitis et al., 2016). Lipid metabolites of the phospholipase A2 pathway were shown to be associated with brain volume in children with CM (Pappa et al., 2015), and high brain volume in paediatric CM has been associated with a poor outcome in CM (Seydel et al., 2015; Sahu et al., 2021b). Furthermore, several of these molecules including kyurenate, 1-methylimidazoleacetate, arachidonic acid and dimethylarginine have been associated with mortality (Gupta S. et al., 2017).

The specificity of markers to CM is crucial, since metabolic changes occur in a range of diseases and could lead to misdiagnosis. Many of the currently identified metabolites are reflective of metabolic changes seen in other infectious diseases prevalent in malaria-endemic areas (Tounta et al., 2021) and/or have also been observed in non-CM malaria (Abdelrazig et al., 2017; Gardinassi et al., 2018; Leopold et al., 2019). Thus, longitudinal studies employing repetitive metabolic measurements over the course of the disease would provide a more accurate representation of metabolic changes and potential diagnostic markers. Indeed, the high sensitivity of metabolomics and the easily accessible samples (i.e., urine, serum, plasma) highlight it as an appropriate route for CM diagnosis. Noting the challenge of employing testing in malaria-endemic regions, running metabolomic analyses on every patient sample is unrealistic. Additionally, while validation of specific levels of metabolite and lipid changes using longitudinal studies could aid this, the costs of these are still high, and therefore remain challenging.

### Potential combinations of biomarkers to increase specificity and accuracy

The combination of specific biomarkers mentioned within this review also show promise in increasing diagnosis specificity and accuracy. IFN-y-related clusters of cytokines, similarly to IFN-y alone, was shown to be associated with MM or SM, but non-specific to CM (Prakash et al., 2006). The same study also investigated a cluster of TNF, TGFβ, IL-10, and IL-1β and found it correlated significantly with CM. A combination of six biomarkers including Ang-2, CXCL10, sFlt-1, PCT, sTREM-1 and sICAM-1 in 2011 was able to predict mortality in Ugandan children with SM and showed 95.7% sensitivity and 88% specificity in differentiating fata and non-fatal cases (Erdman et al., 2011). In addition, the Ang-2/1 ratio has been shown as a robust biomarker of malarial severity (Prapansilp et al., 2013; de Jong et al., 2016). Combination of CXCL4 and CXCL10 have also been shown to predict risk of fatal CM (Wilson et al., 2011). ROC curve analysis demonstrated their ability to differentiate CM non-survivors from those with mild malaria (P<0.0001) and from CM survivors (P<0.0001), with an AUC of 100% (Wilson et al., 2011). However, further research with larger sample size is needed. Similarly, combinations of cytokines and proteins, detection of multiple miRNAs which show promise may prove more specific and accurate than single markers alone (Gupta and Wassmer, 2021). Finally, simultaneously evaluating parasite biomass, number of Group A–EPCR var transcripts, and degree of thrombocytopenia, may offer insight into disease prognosis (Sahu et al., 2021a).

## Conclusions

Over the last decade, many studies have focused on a wide range of candidate biomarkers to improve the diagnosis of CM. While some show great promise, such as antibody-based detection of cytokine and endothelial dysfunction signature panels and miRNAs, these still require extensive research and development to allow their deployment to malarious areas. In addition to their encouraging accuracy, focus should be made to ensure these new tools are minimally invasive, cost-effective, accessible, easily measured and produce rapid results with high sensitivity and specificity.

## Author contributions

HG and SW designed and conceptualized the manuscript. PM, EW and HG carried out the literature search and together with SW generated the first draft of the manuscript. All authors contributed to the article and approved the submitted version.
